# Chronic Thromboembolic Pulmonary Hypertension in a Patient With Systemic Lupus Erythematosus and Antiphospholipid Syndrome: A Case of Surgical Management With Pulmonary Endarterectomy

**DOI:** 10.7759/cureus.106075

**Published:** 2026-03-29

**Authors:** Adan Becerril Ponce, Jonhatan M Cota-Arce, Jesus A Arenas-Lugo, Luis A Morales-Díaz, Hector F Ortiz-Almeyda

**Affiliations:** 1 Cardiothoracic Surgery, Instituto Mexicano del Seguro Social, Mexico City, MEX; 2 Cardiology, Instituto Mexicano del Seguro Social, Mexico City, MEX

**Keywords:** antiphospholipid syndrome, cardiopulmonary bypass, chronic thromboembolic pulmonary hypertension, circulatory arrest, pulmonary endarterectomy, pulmonary hypertension, right ventricular dysfunction, systemic lupus erythematosus, thromboembolism

## Abstract

We present the case of a 22-year-old female with a history of systemic lupus erythematosus and antiphospholipid syndrome, complicated by recurrent deep vein thrombosis of the lower extremities and pulmonary embolism, who subsequently developed chronic thromboembolic pulmonary hypertension. The patient presented with progressive dyspnea, ultimately limiting daily activities, and was managed with long-term anticoagulation and pulmonary vasodilator therapy.

Diagnostic workup, including imaging and hemodynamic assessment, revealed multiple bilateral segmental perfusion defects, right heart dilation, and significantly elevated pulmonary pressures with increased pulmonary vascular resistance, consistent with operable chronic thromboembolic disease.

The patient underwent pulmonary endarterectomy via median sternotomy under cardiopulmonary bypass with deep hypothermia and intermittent total circulatory arrest. Two periods of circulatory arrest, of 19 and 18 minutes, were utilized during the procedure. Organized thromboembolic material was successfully removed from both pulmonary arteries, achieving adequate endarterectomy planes without major intraoperative complications.

In the immediate postoperative period, the patient remained hemodynamically stable, with expected improvement in right ventricular afterload and pulmonary hemodynamics. This case highlights the importance of recognizing chronic thromboembolic pulmonary hypertension in young patients with autoimmune disease and recurrent thrombotic events, as well as the role of pulmonary endarterectomy as a potentially curative treatment when performed in specialized centers.

## Introduction

Chronic thromboembolic pulmonary hypertension is a distinct form of pulmonary vascular disease, characterized by persistent obstruction of the pulmonary arteries by organized thromboembolic material, leading to progressive elevation of pulmonary vascular resistance and right ventricular overload [1]. Although it represents a potentially curable cause of pulmonary hypertension, it often remains underdiagnosed, particularly in younger patients and in those with complex underlying conditions [1].

Autoimmune diseases, particularly systemic lupus erythematosus and antiphospholipid syndrome, are well-recognized risk factors for recurrent thromboembolic events and may contribute to the development of chronic thromboembolic pulmonary hypertension [2]. In these patients, the coexistence of a prothrombotic state and systemic inflammation poses diagnostic and therapeutic challenges, often requiring a multidisciplinary approach [2].

Pulmonary endarterectomy remains the treatment of choice in patients with operable disease, offering significant hemodynamic and clinical improvement when performed in experienced centers [3]. The procedure requires cardiopulmonary bypass, deep hypothermia, and periods of total circulatory arrest to allow adequate visualization and substantial removal of organized thromboembolic material from the pulmonary arterial tree [3].

We report the case of a young woman with systemic lupus erythematosus and antiphospholipid syndrome, who developed chronic thromboembolic pulmonary hypertension and was successfully treated with pulmonary endarterectomy.

## Case presentation

A 22-year-old female with a history of systemic lupus erythematosus, diagnosed in 2023, and antiphospholipid syndrome, complicated by prior venous thromboembolism and pulmonary embolism, was evaluated for progressive dyspnea. Her medical history was significant for pleural and pericardial involvement, as well as recurrent thrombotic events requiring long-term anticoagulation. She was receiving mycophenolic acid, low-dose prednisone, and hydroxychloroquine for lupus management, in addition to oral anticoagulation and pulmonary vasodilator therapy.

Over the preceding months, the patient reported worsening exertional dyspnea, eventually limiting her daily activities. At the time of admission, she was hemodynamically stable, with no chest pain but functionally limited by shortness of breath with minimal exertion.

Transthoracic echocardiography demonstrated preserved left ventricular systolic function, with a left ventricular ejection fraction of 65%. The right ventricle was dilated, with preserved systolic function, and both atria were enlarged, with severe right atrial dilation. Severe tricuspid regurgitation was observed, with an estimated pulmonary artery systolic pressure of 72 mmHg. The pulmonary artery was dilated, and no pericardial effusion was noted.

**Figure 1:**
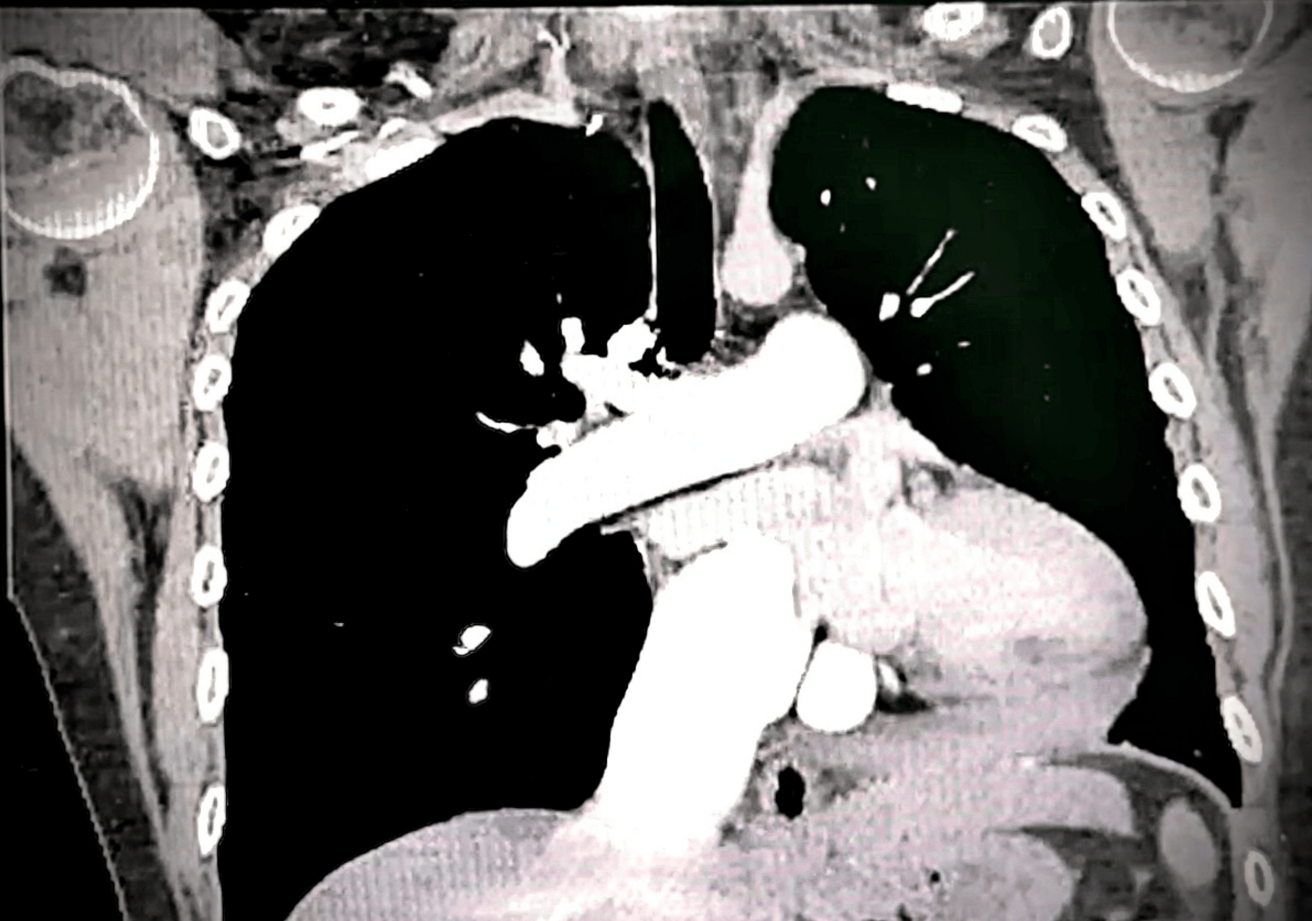
Computed tomography angiography demonstrating chronic thromboembolic pulmonary disease Coronal contrast-enhanced computed tomography of the chest shows the absence of vascular flow in the left lower lobe pulmonary artery, consistent with chronic occlusion. The main pulmonary artery appears dilated, with reduced distal vascularization in the affected territory - findings compatible with chronic thromboembolic pulmonary hypertension.

**Figure 2:**
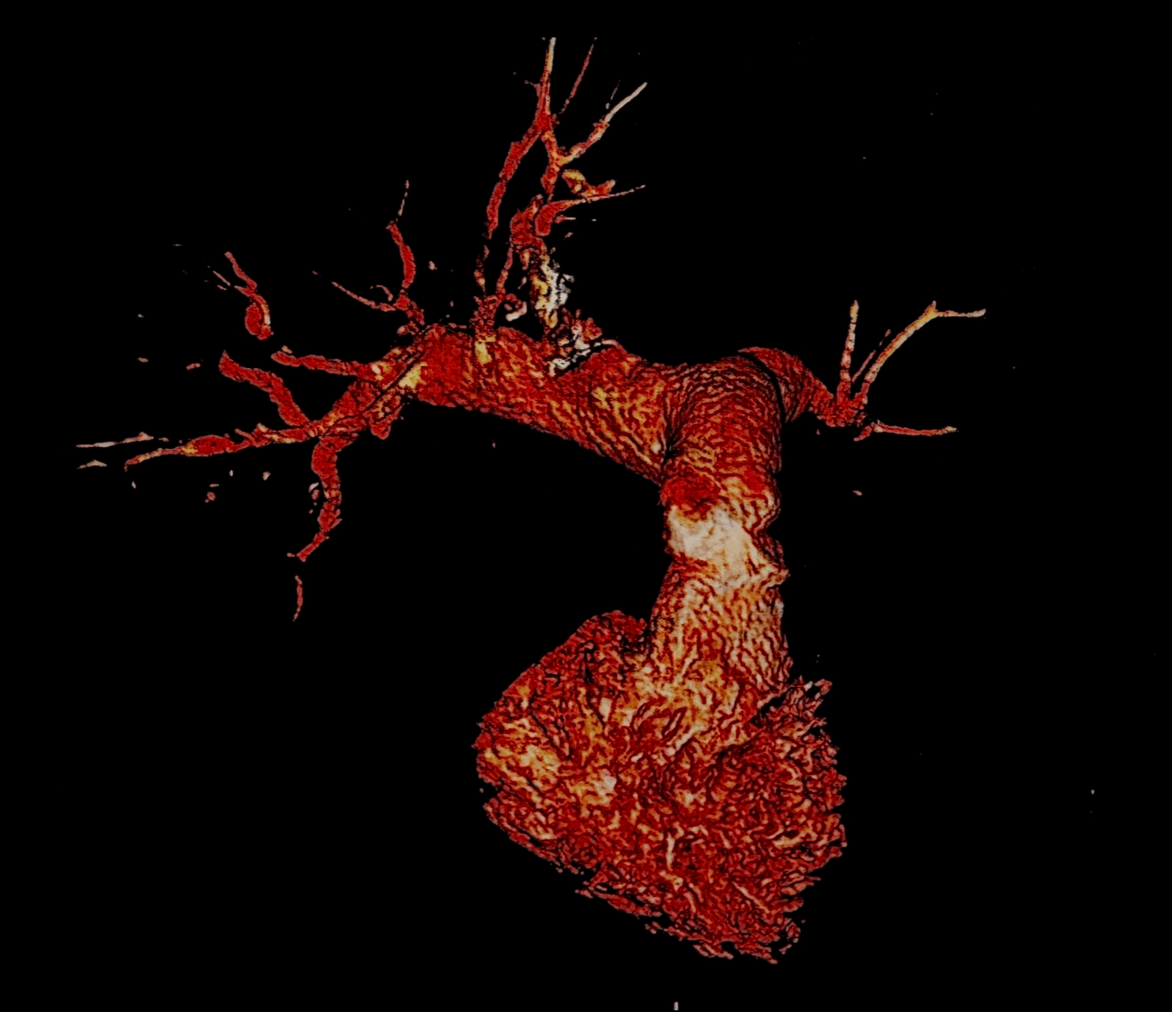
Extracted material reproduced the morphology of the pulmonary arterial tree, confirming adequate endarterectomy planes Gross specimen of organized thromboembolic material extracted during pulmonary endarterectomy, demonstrating a branching cast of the pulmonary arterial tree. The specimen reflects chronic fibrotic transformation of thromboemboli, with extension into segmental and subsegmental branches - consistent with chronic thromboembolic pulmonary hypertension.

Ventilation-perfusion imaging revealed multiple bilateral segmental perfusion defects, consistent with chronic thromboembolic disease. Computed tomography angiography demonstrated the absence of vascular flow to the left lower lobe, suggestive of chronic occlusion (Figure 1). Three-dimensional reconstruction confirmed irregularity and distal pruning of the pulmonary arterial tree (Figure 2).

Right heart catheterization confirmed severe pulmonary hypertension, with pulmonary artery pressures of 92/42 mmHg (mean 56 mmHg) and a pulmonary vascular resistance of 7 Wood units.

**Figure 3:**
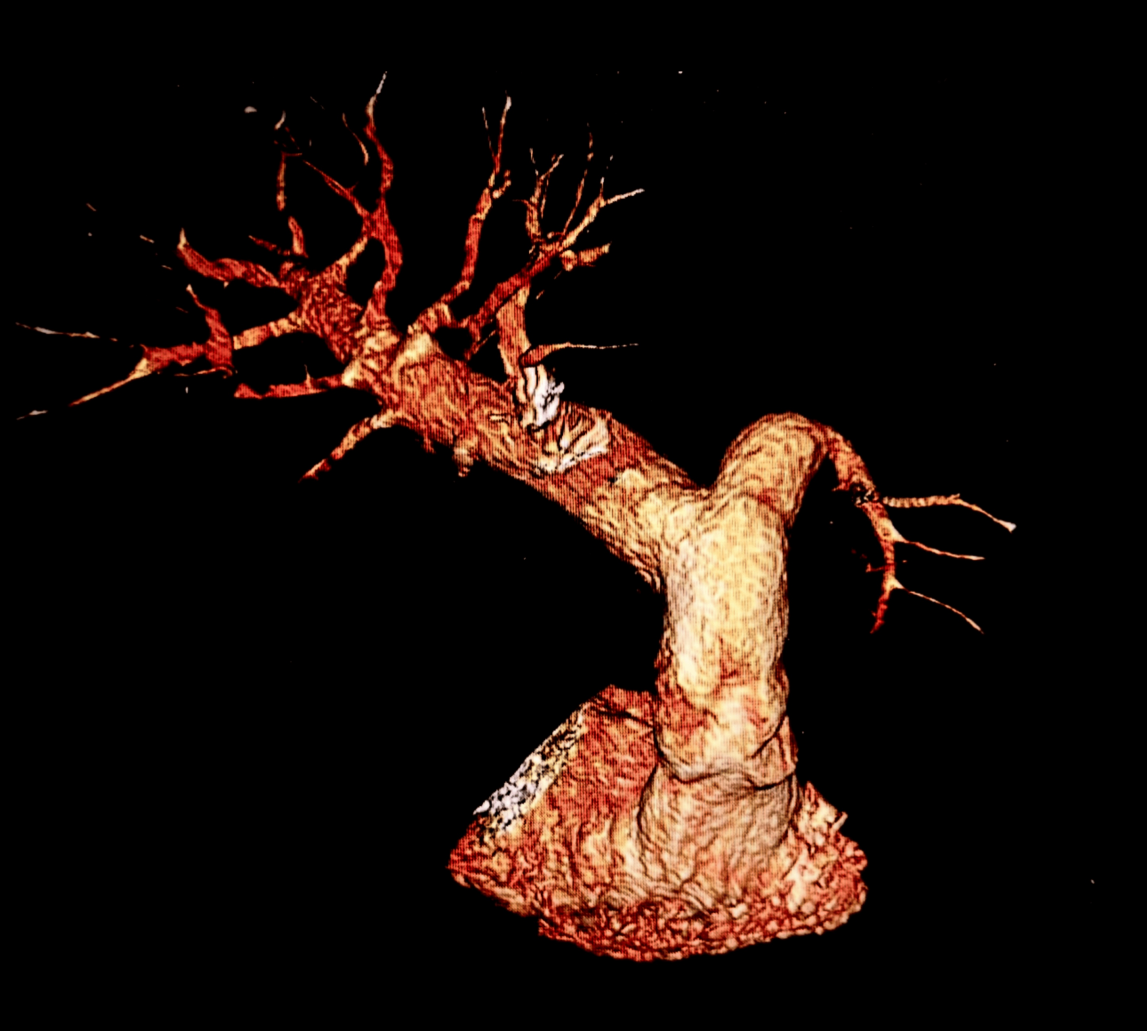
Three-dimensional computed tomography reconstruction of the pulmonary arteries Three-dimensional reconstruction from contrast-enhanced computed tomography showing the pulmonary arterial tree. Irregularity and narrowing of segmental branches, with areas of reduced distal perfusion, are observed - consistent with chronic thromboembolic pulmonary hypertension.

**Figure 4:**
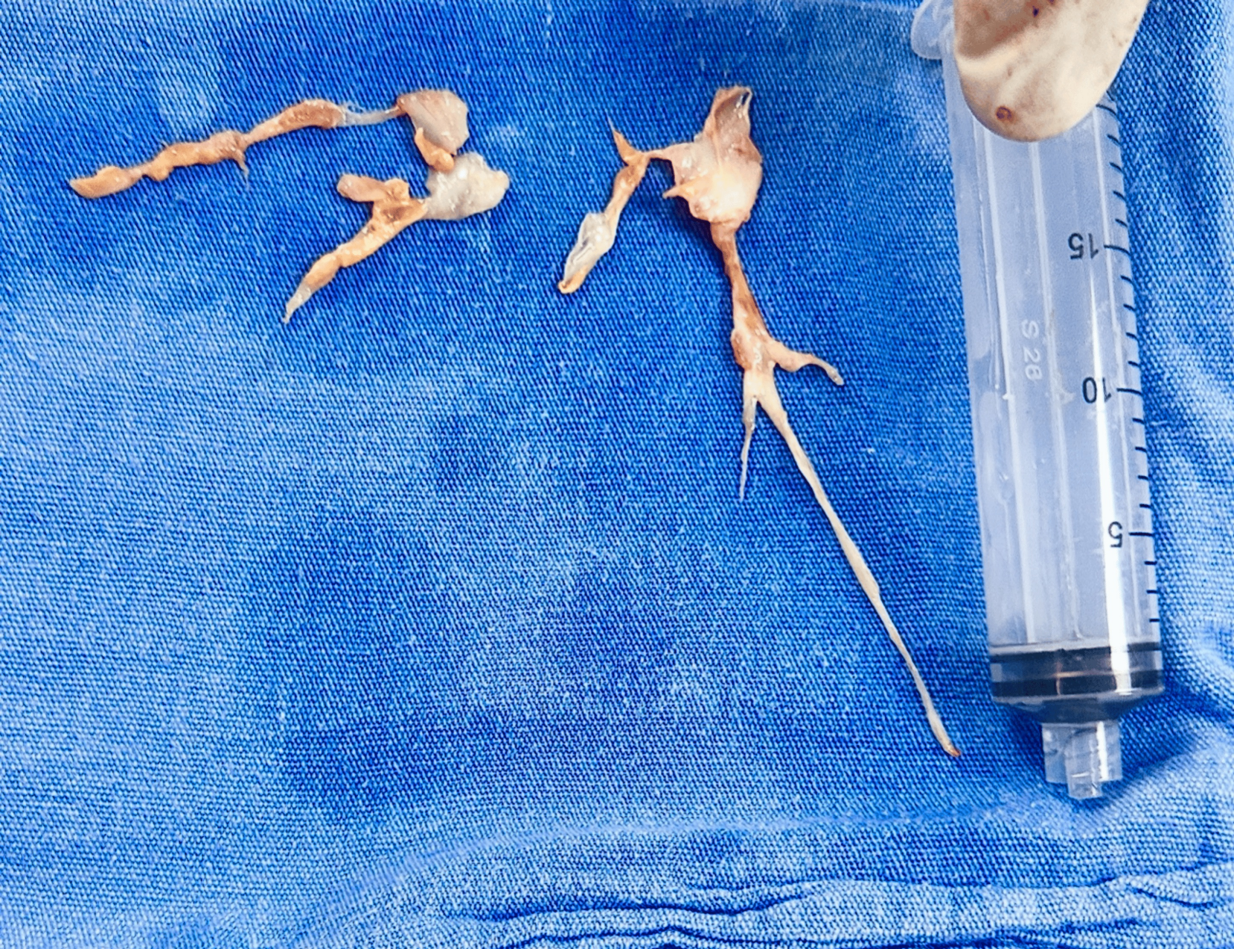
Organized thromboembolic material removed during pulmonary endarterectomy Intraoperative photograph showing organized thromboembolic material removed from the pulmonary arteries during pulmonary endarterectomy. The specimen demonstrates fibrotic transformation, with branching extensions corresponding to segmental arterial involvement - consistent with chronic thromboembolic pulmonary disease.

Given the clinical, imaging, and hemodynamic findings, a diagnosis of operable chronic thromboembolic pulmonary hypertension was established. The patient underwent pulmonary endarterectomy. Intraoperative findings included removal of organized thromboembolic material, including fibrotic fragments, and a branching cast of the pulmonary arterial tree (Figures 3-4).

## Discussion

Chronic thromboembolic pulmonary hypertension represents a potentially curable form of pulmonary hypertension when appropriately diagnosed and treated [1,3]. It results from incomplete resolution of thromboembolic events and subsequent organization of thrombotic material within the pulmonary arterial tree, leading to progressive increases in pulmonary vascular resistance and right ventricular overload [4].

The association between systemic lupus erythematosus and antiphospholipid syndrome significantly increases the risk of recurrent thromboembolic events [2]. In this context, a persistent prothrombotic state, combined with endothelial dysfunction, contributes to chronic vascular obstruction and disease progression.

Pulmonary endarterectomy remains the gold standard treatment for patients with surgically accessible disease, providing substantial hemodynamic improvement and potential cure [3,5]. In experienced centers, the procedure is associated with low perioperative mortality, significant reductions in pulmonary vascular resistance, and improvement in functional status and long-term survival [5].

Despite these favorable outcomes, perioperative management remains critical. One of the most relevant complications is reperfusion lung injury, which may require advanced supportive measures, including mechanical ventilation strategies or extracorporeal support in severe cases [3,6]. Long-term anticoagulation is essential in patients with antiphospholipid syndrome to prevent recurrent thrombosis. Although representative echocardiographic and ventilation-perfusion imaging were not included, the diagnosis was supported by consistent multimodal imaging and hemodynamic findings.

This case highlights the importance of maintaining a high index of suspicion for chronic thromboembolic pulmonary hypertension in young patients with autoimmune disease and recurrent thrombotic events. Early recognition and referral to specialized centers are crucial, as surgical treatment can significantly alter the natural history of the disease.

## Conclusions

Chronic thromboembolic pulmonary hypertension should be considered in young patients with autoimmune disease and a history of recurrent thromboembolic events. This case illustrates that, despite severe pulmonary hypertension, pulmonary endarterectomy can be safely performed, with favorable outcomes when the disease is surgically accessible. Early diagnosis and referral to specialized centers are essential, as timely surgical intervention may significantly improve hemodynamics and clinical status, altering the natural course of the disease.
